# 504. Case SEries: Nasal Antimicrobial Photodisinfection (APDT) as Treatment Protocol for Asymptomatic and Early Stage COVID-19 Patients

**DOI:** 10.1093/ofid/ofab466.703

**Published:** 2021-12-04

**Authors:** Jack Kolenda, Josepmaria Argemi

**Affiliations:** 1 University of Toronto, Oakville, Ontario, Canada; 2 Universidad de Navarra, Pamplona, Navarra, Spain

## Abstract

**Background:**

Targeted reduction of SARS-CoV-2 viral load in the nose may mitigate the severity of lower tract respiratory disease as well as reduce hospitalization and mortality rates. Nasal Photodisinfection has been deployed for 10 years in Canadian hospitals reducing post-surgical infections. The objective of thiswork was to demonstrate effectiveness of APDT in early stage COVID-19 and asymptomatic carriers.

**Methods:**

A cohort of 40 COVID-19 positive patients were treated with nasal photodisinfection (Steriwave) at a private clinic. All patients were previously identified by PCR as SARS-CoV-2 positive and admitted into the treatment cohort. BD rapid antigen nares testing was used before and after Photodisinfection treatment. Of the 40 patients, 13 were female and 27 were male. Age range was 9- 56 years of age. Treatment involved 3-4 applications of photosensitizer and 16-24 minutes per patient of treatment time. Patients were followed up within 24 hours, 48 hours as well as day 5 and 6 and day 10/11. Patients filled out a COVID-19 score card.

**Results:**

Results demonstrated APDT was capable of significant and rapid viral load reduction in COVID-19 carriers. 100% of patients were converted from positive rapid antigen test to negative. 60% of patients reported fever resolution within 24 hours. Fever resolution occurred in 100% of patients within 48hours. Moreover, results demonstrated accelerated resolution of COVID-19 symptoms and significantly improved mental health benefits from reduction of COVID-19 related stress and anxiety. None of the patients experienced severe symptoms and no patients were hospitalized. Safety outcomes demonstrated no patient safety issues with only minor transient side effects (rhinorrhea, sneezing) observed. Moreover, the treatment procedure was pain-free and well tolerated by all patients.

**Conclusion:**

Photodisinfection-based nasal decolonization anti-viral efficacy was demonstrated with improved outcomes for all patients treated in this case series. Significant rapid viral load reduction was confirmed by rapid antigen tests in all patients. More clinical studies are warranted in support of Photodisinfection based therapy for upper respiratory infections such as COVID-19.

**Disclosures:**

**All Authors**: No reported disclosures

